# Cerebellar Transcranial Magnetic Stimulation: The Role of Coil Geometry and Tissue Depth^☆^

**DOI:** 10.1016/j.brs.2014.04.009

**Published:** 2014-09

**Authors:** Robert M. Hardwick, Elise Lesage, R. Chris Miall

**Affiliations:** a Department of Physical Medicine and Rehabilitation, Johns Hopkins University, Baltimore, MD, USA; b Behavioural Brain Sciences, School of Psychology, University of Birmingham, Birmingham, UK

**Keywords:** Cerebellum, TMS, Cerebello brain inhibition, Cerebellar brain inhibition, TMS coil geometry, Deep TMS, Figure-of-eight coil, Double cone coil, Batwing coil

## Abstract

**Background:**

While transcranial magnetic stimulation (TMS) coil geometry has important effects on the evoked magnetic field, no study has systematically examined how different coil designs affect the effectiveness of cerebellar stimulation.

**Hypothesis:**

The depth of the cerebellar targets will limit efficiency. Angled coils designed to stimulate deeper tissue are more effective in eliciting cerebellar stimulation.

**Methods:**

Experiment 1 examined basic input–output properties of the figure-of-eight, batwing and double-cone coils, assessed with stimulation of motor cortex. Experiment 2 assessed the ability of each coil to activate cerebellum, using cerebellar-brain inhibition (CBI). Experiment 3 mapped distances from the scalp to cerebellar and motor cortical targets in a sample of 100 subjects' structural magnetic resonance images.

**Results:**

Experiment 1 showed batwing and double-cone coils have significantly lower resting motor thresholds, and recruitment curves with steeper slopes than the figure-of-eight coil. Experiment 2 showed the double-cone coil was the most efficient for eliciting CBI. The batwing coil induced CBI only at higher stimulus intensities. The figure-of-eight coil did not elicit reliable CBI. Experiment 3 confirmed that cerebellar tissue is significantly deeper than primary motor cortex tissue, and we provide a map of scalp-to-target distances.

**Conclusions:**

The double-cone and batwing coils designed to stimulate deeper tissue can effectively stimulate cerebellar targets. The double-cone coil was found to be most effective. The depth map provides a guide to the accessible regions of the cerebellar volume. These results can guide coil selection and stimulation parameters when designing cerebellar TMS studies.

## Highlights

• Properties of a figure-of-eight, batwing and double-cone TMS coil were compared.
• Coil type affected the input–output properties of TMS over M1.
• Only the double-cone coil provided reliable cerebellar stimulation.
• MRI data show the cerebellum is deep, and may require deeper-reaching TMS coils.
• These data can inform the interpretation and design of cerebellar TMS studies.

## Introduction

Transcranial magnetic stimulation (TMS) is a promising technique to probe cerebellar function [1], but the parameters that underlie effective cerebellar stimulation are incompletely understood. The efficacy of TMS is determined by coil geometry, stimulus intensity, and the depth of the targeted tissue [2], [3], [4], [5]. To better understand how TMS might be used to target the cerebellum, we undertook a comparison of different coil types, manipulated stimulus intensity to measure recruitment curves, and compared the depth of several cerebellar stimulation sites from the scalp.

An important challenge is that the cerebellum itself is by-and-large silent to the effects of TMS, making it difficult to directly monitor the effectiveness of the stimulation. The effects of cerebellar stimulation are therefore typically quantified with cerebellar brain inhibition (CBI; 6–11). CBI takes advantage of the cerebellum's inhibitory connections with primary motor cortex, applying a conditioning pulse of TMS to the cerebellum prior to stimulating the primary motor cortex (M1). The resulting motor evoked potentials (MEPs) are typically smaller than those elicited by stimulating M1 alone, reflecting the inhibitory influence of the cerebellum. CBI therefore provides a measure of cerebellar cortical excitation, at least for those cerebellar territories that project to the motor cortex via a direct cerebellar-nuclear-thalamo-cortical route [9].

Different TMS paradigms have used different coil types to stimulate the cerebellum [6], [7], [8], [9], [10], [11], [12], [13], [14], [15], [16], [17], [18], [19], [20], but the properties of these different coils types (e.g. their input–output properties and effectiveness in stimulating the cerebellum) have not been systematically addressed in vivo. The flat figure-of-eight coil is known for its ability to produce focal stimulation, and is frequently used to stimulate relatively superficial neocortical targets [21]. In contrast, the angled double-cone and batwing coils have been designed to stimulate deeper-lying regions [4], [22]. However, studies employing the CBI indicate a relatively strong stimulus (up to 80% of maximal stimulator output) is required to effectively stimulate the cerebellum [6]. This indicates that in relation to neocortical tissue, where activation thresholds can be <40% of maximal stimulator output (MSO), cerebellar tissue is either deeper, less excitable, or both. If it is indeed a deeper-lying target, coils designed for deeper stimulation might be required. However, both figure-of-eight and double-cone coils are used for cerebellar TMS (e.g. 6–20), although the majority of studies using CBI have used a double-cone coil to stimulate the cerebellum [6], [7], [8], [9], [10], [11]. Indeed, one of the first papers reporting CBI found that a double-cone coil provided reliable CBI effects in all participants examined, while a figure-of-eight coil did not [10]. However, this effect was not systematically examined – only four of the eight participants were tested using both coil types. Surprisingly, several studies applying repetitive TMS protocols to the cerebellum have used figure-of-eight coils [15], [16], [17], [18], even when targeting the same areas that apparently require stimulation with a double-cone coil to elicit consistent CBI [15], [17]. These differences in coil choice are not trivial, as modeling studies demonstrate that coil geometry determines important factors such as the depth, width and relative intensity of the electric field generated in the brain [5].

Here we report experiments addressing the effectiveness of cerebellar TMS. We specifically focused on the effects of coil geometry and examined the properties of three coil designs: the figure-of-eight, batwing and the double-cone coils. We relate threshold differences to the depth of primary motor cortex and several often-used cerebellar targets. In Experiment 1 the input–output properties of each coil were determined across a range of intensities when targeting the hand representation in primary motor cortex. Recruitment curves and resting motor threshold (RMT) of these three TMS coil designs were compared. Based on modeling evidence, we hypothesized that coils with angled wings (designed to stimulate deeper targets, i.e. the batwing and especially the double-cone coil; 5) would be able to stimulate the motor cortex most efficiently. This would be reflected by lower RMTs and steeper recruitment curves than those for the figure-of-eight coil. Experiment 2 then assessed the ability of these three coils to effectively stimulate the cerebellum, as evidenced by CBI, at four different intensities of stimulation. We hypothesized that coils designed to stimulate deeper targets would be more efficient in eliciting CBI than the figure-of-eight coil. We also measured participant's self-reported discomfort during stimulation with each coil type. Finally, Experiment 3 assessed the depth of motor cortical and cerebellar targets based on structural MR images from 100 healthy young participants. We provide a map of the depths from the scalp to selected cerebellar targets that can guide future experimental design.

## Methods

### Participants

21 participants gave written informed consent to partake in the study, which was approved by the local ethics panel at the University of Birmingham. Experiment 1 examined responses from 13 participants (mean age ± SD: 23 ± 3, 9 female, 12 right handed). Experiment 2 examined responses from 15 participants (mean age ± SD: 22 ± 2, 9 female, 13 right handed). Seven participants took part in both experiments 1 and 2. Experiment 3 used 100 high-resolution structural images (MPRAGE sequence, resolution 1 × 1 × 1 mm) from 100 subjects (50 male, ages 18–30) from a publicly available database [23].

### Materials

Experiments 1 and 2 used three coils; a figure-of-eight (type no. 3217-00), batwing (type no. 15411-00), and double-cone coil (type no. 9902-00; all by The Magstim Company, Whitland, Wales). Two key differences exist between these coils. Firstly the angle of the windings of the coils differs, being 180° for the figure-of-eight coil, and closer to 135° for the batwing and double-cone coils. Secondly, the diameter of the wings differs between coils, being 70 mm for the figure-of-eight and batwing coils, and 90 mm for the double-cone coil. TMS was delivered using Magstim Rapid and Rapid2 devices, both with identical biphasic output profiles (personal correspondence, the Magstim Co.). Electromyograms were recorded from the first dorsal interosseus (FDI) muscle of the dominant hand, and the motor hotspot and RMT for M1 were determined according to well-established and previously documented procedures (see Supplementary Materials).

### Experiment 1: recruitment curves

Recruitment curves for each coil were measured according to a previously documented procedure [24], [25], [26]. TMS pulses were delivered over the left primary motor cortex at eight different intensities, ranging from 90% to 160% of RMT in 10% increments. A custom written computer script for CED's Signal data collection software (CED, Cambridge, UK) was used to collect MEPs in a pseudorandom order, such that one of each of the eight stimulus intensities was applied every eight trials. The time between pulses was varied between 5 and 8 s, in order to avoid repetitive TMS effects and predictability of the stimulus. Ten trials were collected at each stimulus intensity, yielding a total of 80 MEPs for each coil.

### Experiment 2: cerebellar-brain inhibition

Cerebellar activation was assessed using a previously established CBI protocol [6], [7], [8], [9], [10], [11]. A figure-of-eight coil was used to stimulate the motor cortex, eliciting MEPs from the FDI muscle with peak-to-peak amplitudes of approximately 1 mV. Cerebellar stimulation was applied via a coil positioned 1 cm inferior and 3 cm lateral to the inion (referred to as 3L1I) on the contralateral side of the head corresponding to the participant's dominant hand [13], [20].

CBI was measured by collecting blocks of 40 MEPs. Each block consisted of 20 conditioned MEPs (collected 5 ms after a TMS conditioning stimulus was delivered to the cerebellum), and 20 control MEPS. Conditioned and control MEPs were collected in a random interleaved order. This procedure was repeated for each of the coil designs at fixed conditioning stimulus intensities of 65%, 70%, 75% and 80% of maximum stimulator output (MSO). This range of intensities was determined in a pilot experiment where a tolerable range of cerebellar stimulation intensities that could elicit CBI in individual participants was established, and is consistent with the approach employed by Galea et al. [6]. The order in which the coils were assessed was counterbalanced between participants.

TMS stimulation can activate neck muscles, leading to muscle contractions and discomfort [16]. Participants rated the discomfort associated with the highest (80%) and lowest intensity (65%) of TMS experienced with each coil on a scale of 1 (minimal discomfort – “not unpleasant at all”) to 7 (maximum discomfort – “unbearable”). Participants were stimulated with each coil in a counterbalanced manner to prevent order effects.

### Experiment 3: tissue depth at cortical and cerebellar scalp targets

The depth of the tissue being stimulated during TMS is an important factor for effective stimulation [2], [3], [4]. Two of the most frequently used stimulation sites for cerebellar TMS are 3 cm lateral to the inion (3L; [6], [7], [8], [9], [10], [11]) or 3 cm lateral and 1 cm inferior to the inion (3L1I; [12], [13], [20]). Here we determined the depth of the cerebellum from these scalp coordinates in high-resolution structural T1 images of 100 subjects. Cartesian distances to the primary motor cortex hand representation, cerebellar gray matter, and cerebellar hand representations were calculated (see Supplementary Methods).

### Data analysis

#### Experiment 1

Trials revealing background muscle contractions (peak-to-peak EMG activity outside of a 95% confidence interval around the background median in the 200 ms prior to M1 stimulation) were removed [27]. MEP recruitment curves were assessed by fitting regression lines to the approximately linear part of the recruitment curve ([24], [25], [26], i.e. from 90% to 140% of RMT). Slope parameters were estimated for each coil and each participant. RMT and slope estimates were analyzed using repeated measures ANOVAs comparing the three coil designs. Significant main effects were examined using pairwise *t*-tests (Bonferonni corrected alpha of 0.017).

#### Experiment 2

CBI was assessed using a 3 × 4 × 2 repeated measures ANOVA with factors cerebellar coil geometry (figure-of-eight, batwing, double-cone), intensity of cerebellar stimulation (65%, 70%, 75% or 80% of MSO) and trial type (conditioned, control). Trials with high background muscle activity were excluded as in Experiment 1. Planned *t*-tests were conducted based on the *a priori* hypothesis that conditioned MEP amplitudes would be significantly smaller than control MEP amplitudes. We conducted 1-tailed tests (as we expected only inhibitory effects) for each combination of coil and stimulation intensity, controlling for multiple comparisons (Bonferroni corrected alpha *P* < 0.00417). Participant ratings of discomfort during cerebellar stimulation were analyzed using a 3 × 2 repeated measures ANOVA with fixed factors coil type (figure-of-eight, batwing, double-cone) and intensity of stimulation (highest intensity, lowest intensity). Finally, the procedure documented by Fisher et al. [11], was used to assess evidence of direct stimulation of the brainstem (comparing conditioned and unconditioned MEP response latencies; see Supplementary Methods).

#### Experiment 3: tissue depth analysis

The depth of M1 and selected cerebellar targets were estimated in structural scans from 100 subjects. We quantified the shortest Cartesian distances from the scalp to the M1 hand representation, and from scalp locations 3L and 3L1I relative to the inion to cerebellar gray matter, and to the cerebellar hand representations in lobules V and VIII. Tissue boundaries were found using SPM5 segmentation algorithm and the SUIT toolbox [28], [29]. Motor representations were estimated from group fMRI activation clusters ([30]; see Supplementary Methods for details). Because occipital lobe stimulation may in some cases confound the results of cerebellar TMS studies, we also determined whether these shortest distance measurements took a path that directly intersected the occipital cortex.

## Results

### Experiment 1: effects of coil geometry on motor recruitment curves

#### Resting motor thresholds

**Figure 1:**
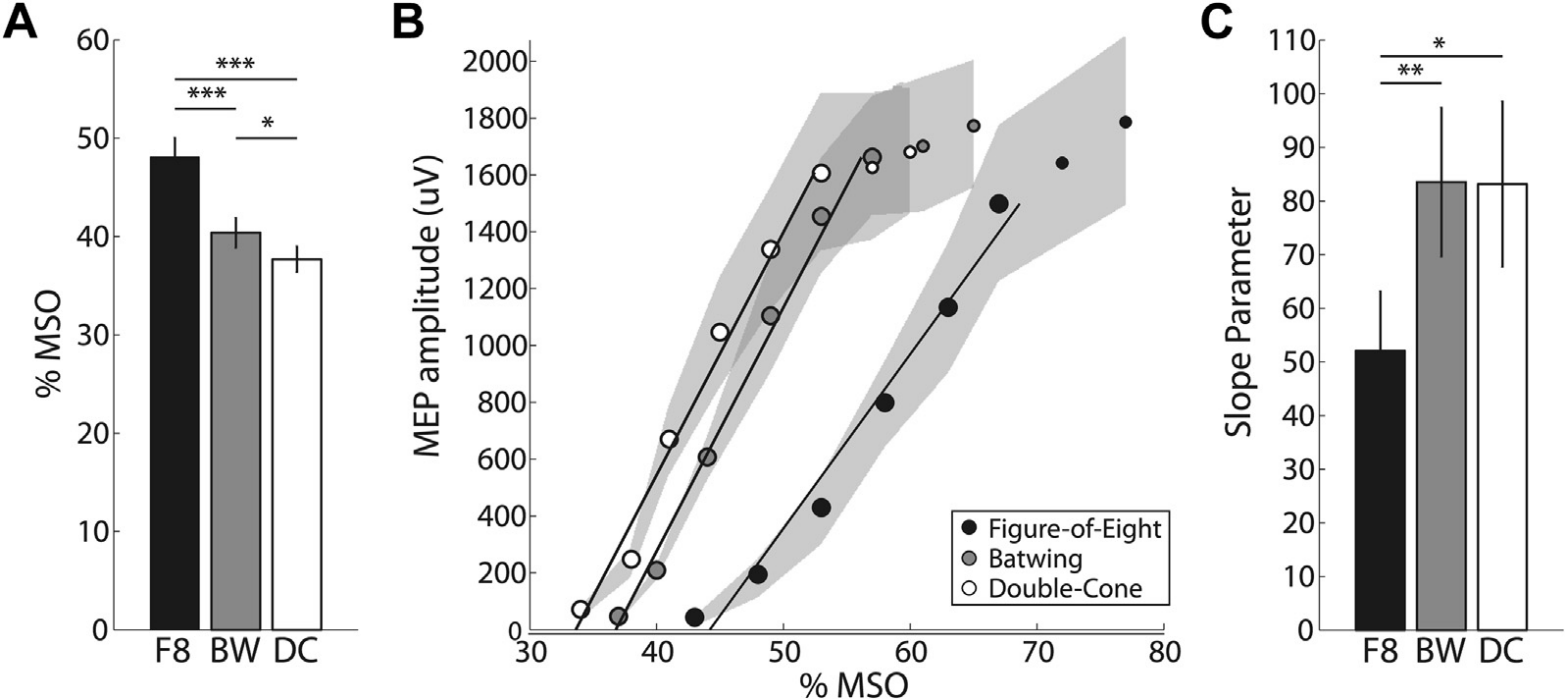
Primary motor cortex excitability as a function of coil geometry. A) Mean intensity (% of MSO) required to achieve motor threshold with each coil design. B) MEP recruitment curves for each coil, with regression lines fit to the approximately linear part of the curve, i.e. 90%–140% of RMT ([24], [25], [26]). Smaller circles indicate points that were not included in the slope estimation. C) Slope parameters for the recruitment curve fits shown in panel B. F8: Figure-of-eight coil, B: Batwing coil, DC: Double-cone coil. Error bars represent ± 1 standard error of the mean (SEM).

RMT over the M1 hotspot was compared for the three coil types examined (see Fig. 1A). 2.4% of trials were removed from the analysis due to muscle preactivation. A repeated measures ANOVA revealed a significant main effect of coil geometry (*F*_2,24_ = 42.0, *P* < 0.001). Bonferroni corrected pairwise comparisons revealed significant differences between each coil, such that RMTs for the figure-of-eight coil were significantly higher than RMTs for the batwing coil (*t*_12_ = 7.5, *P* < 0.001), and the double-cone coil (*t*_12_ = 6.9, *P* < 0.001). RMTs for the batwing coil were also significantly higher than RMTs for the double-cone coil (*t*_12_ = 2.9, *P* = 0.013).

#### Recruitment curves

Regression lines were fit to the approximately linear part of the recruitment curves for each coil (see Fig. 1B). Analysis of the slope parameter estimates (see Fig. 1C) revealed a significant main effect of coil type (*F*_2,24_ = 6.6, *P* = 0.005). Bonferroni corrected pairwise comparisons revealed that MEP recruitment curves were less steep for the figure-of-eight coil compared to the batwing coil (*t*_12_ = 3.1, *P* = 0.009), and the double-cone coil (*t*_12_ = 2.9, *P* = 0.014). Recruitment curve slopes for the batwing and double-cone coils did not differ (*t*_12_ = 0.0, *P* = 0.96). That is, increasing stimulus input by 10% has a greater effect on MEP amplitude with batwing or double-cone coil than with a figure-of-eight coil.

### Experiment 2: effects of coil geometry on cerebello-brain inhibition

#### Cerebellar-brain inhibition

**Figure 2:**
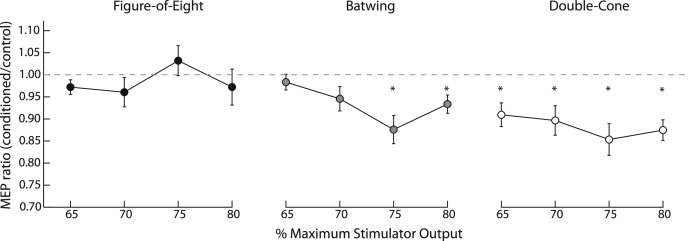
Cerebello-brain inhibition ratios for the three different coil designs. Circles present mean group data for each block of MEPs collected, with the data normalized by dividing the mean conditioned MEP amplitude by the mean control MEP amplitude. *indicates a significant difference between the conditioned and control MEP amplitude (all Bonferroni corrected one tailed *t*-test, *P* < 0.00417).

Figure 2 presents MEP ratios (mean conditioned amplitudes divided by mean control amplitudes). 2.2% of trials were removed from the analysis due to muscle preactivation. The hypothesized three-way interaction between coil type, stimulation intensity and pulse conditioning was statistically significant (*F*_6,84_ = 2.2, *P* = 0.047). Planned one tailed *t*-tests compared control and conditioned MEP amplitudes for each coil and intensity. CBI was not present (i.e. conditioned MEP amplitudes did not differ from control MEP amplitudes) when the cerebellum was stimulated using a figure-of-eight coil at any of the intensities applied (65–80% MSO), or with a batwing coil at the lower intensities of 65% and 70% of MSO (all *t* < 2.1, *P* > 0.05). Significant CBI effects (i.e. conditioned MEP amplitudes smaller than control MEP amplitudes) were present when the cerebellum was stimulated using the batwing coil at the higher intensities of 75% and 80% MSO, and with the double-cone coil at all of the intensities applied here (65–80% MSO; all *t* > 3.4, *P* < 0.00417). The control for direct brainstem effects revealed no difference between latencies for control and conditioned MEPs (all *F* < 1.5, *P* > 0.25), indicating that direct stimulation of the brainstem did not confound results (see Supplementary Materials).

#### Perceived discomfort of cerebellar stimulation

**Figure 3:**
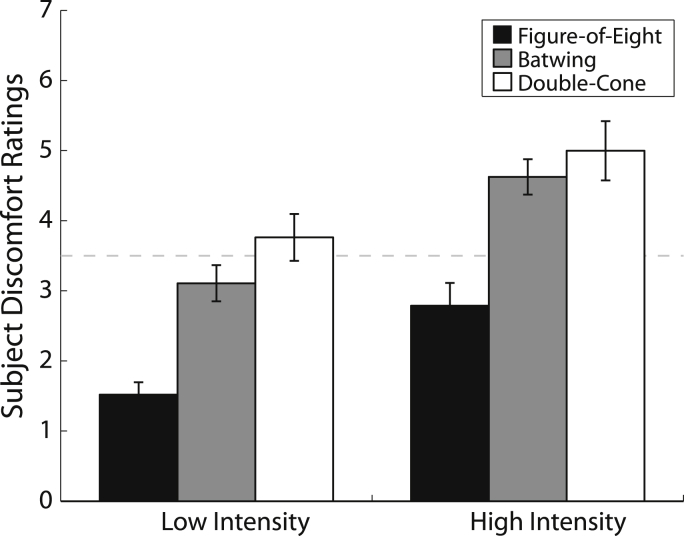
Perceived discomfort of cerebellar TMS. Ratings were on a scale ranging from 1 (“No discomfort”) to 7 (“Unbearable”). Error bars represent ± 1 SEM. The dashed line separates conditions where CBI was not (below) and was (above) induced.

The repeated-measures ANOVA revealed a main effect of Coil (*F*_1.7, 46.1_ = 26, *P* < 0.001) and Stimulus intensity (*F*_1,37.7_ = 32, *P* < 0.001). Stimulation with the figure-of-eight coil was deemed more comfortable than with the batwing coil (*t*_27_ = 8.32, *P* < 0.001, Bonferroni corrected) and double-cone coil (*t*_27_ = 8.48, *P* < 0.001, Bonferroni corrected), which did not differ from each other (*t*_27_ = 1.72, *P* = 0.192, Bonferroni corrected; see Fig. 3). All coils were deemed more comfortable at lower intensities of stimulation.

### Experiment 3: tissue depth analysis

**Figure 4:**
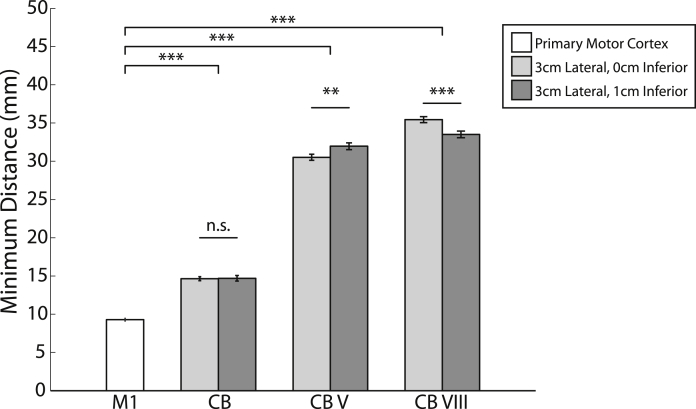
Depth of different tissue types in relation to target scalp locations. CB: cerebellar gray matter, CB V: Cerebellar lobule V hand representation, CB VIII: Cerebellar lobule VIII hand representation, Light gray: scalp location 3L, Darker gray: scalp location 3L1I. Error bars represent ± 1 SEM.

**Figure 5:**
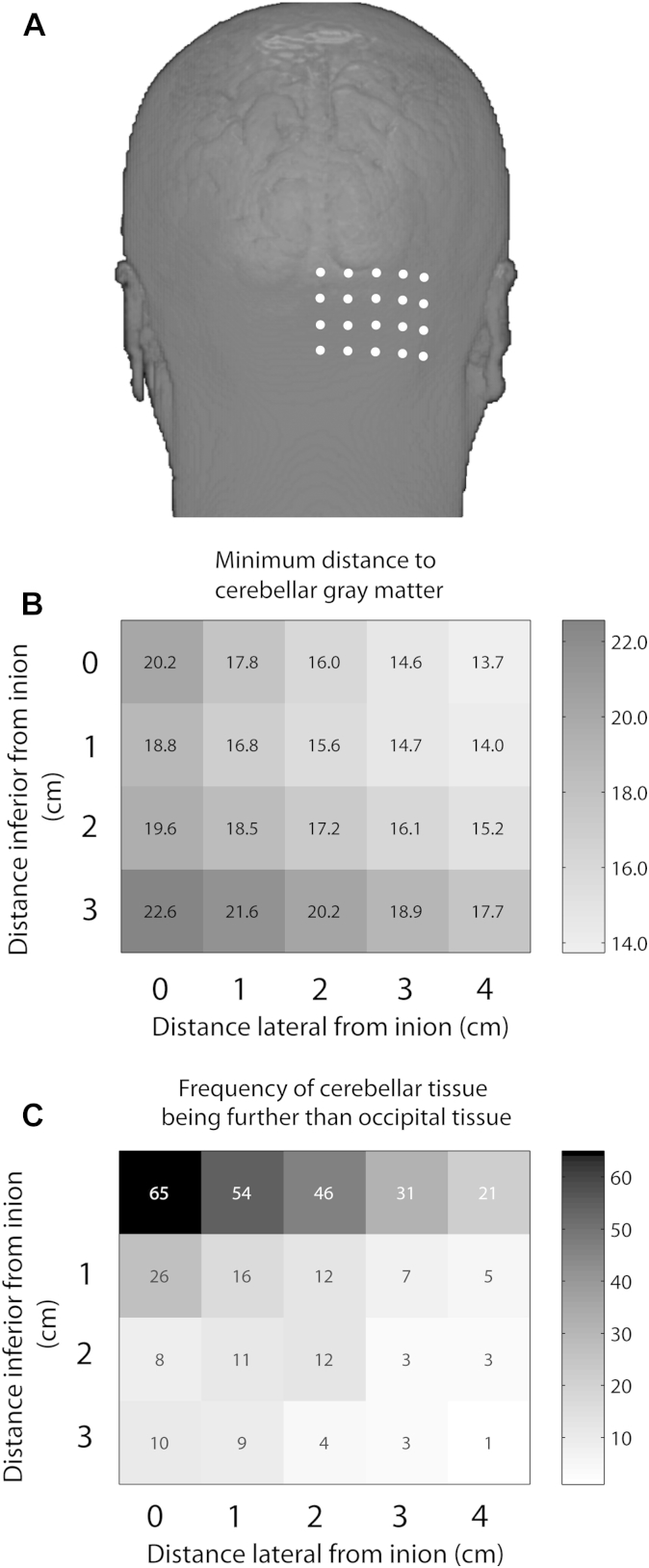
Data obtained from structural MRI scans from a sample of 100 subjects. A) For each subject a grid of scalp positions at 1 cm intervals was calculated relative to the inion. B) Cartesian distances from the grid of scalp positions to the closest gray matter (mm). Lighter colors indicate shorter distances. C) Number of participants in which the shortest path to cerebellar tissue took a path through the occipital cortex. Lighter colors indicate positions where this path was less likely to pass through occipital tissue.

Figure 4 illustrates the shortest distance from the scalp to M1, and that from the two most frequently used cerebellar stimulation sites to cerebellar gray matter and cerebellar motor representations in a sample of 100 individuals. Results showed that the distance from the scalp to the primary motor cortex (9.3 ± 1.6 mm) was significantly shorter than the distance from the scalp to cerebellar gray matter for coordinates 3L (14.6 ± 2.6 mm, *t*_99_ = 17.7, *P* < 0.001) and 3L1I (14.7 ± 3.6 mm, *t*_99_ = 13.6, *P* < 0.001). The primary motor cortex was also less deep than the cerebellar hand representations in lobule V (30.5 ± 4.0 mm from scalp location 3L, *t*_99_ = 48.3, *P* < 0.001, and 32.0 ± 4.5 mm from 3L1I, *t*_99_ = 46.7, *P* < 0.001) and lobule VIII (35.4 ± 3.9 mm from 3L, *t*_99_ = 60.0, *P* < 0.001, and 33.5 ± 4.3 mm from 3L1I, *t*_99_ = 50.6, *P* < 0.001). Distances were notably longer for the two cerebellar motor representations than for cerebellar gray matter (see Fig. 4). Comparisons indicated no significant differences in depth between the 3L and 3L1I scalp locations when targeting cerebellar gray matter (difference = 0.05 mm; *t*_99_ = 0.2, *P* = 0.81). The 3L position was significantly closer than the 3L1I position when targeting the lobule V hand representation (difference = 1.45 mm; *t*_99_ = 7.9, *P* < 0.001), and significantly further when targeting the lobule VIII hand representation (difference = 1.94 mm; *t*_99_ = 12.4, *P* < 0.0001). When considering all assessed scalp locations (see Fig. 5A), the absolute distance to cerebellar gray matter tissue was shortest for 3 cm and 4 cm lateral to the inion (3L: 14.6 ± 2.6 mm; 4L: 13.7 ± 2.8 mm), and for the same locations 1 cm inferior to the inion (3L1I: 14.7 ± 3.6 mm; 4L1I: 14.0 ± 3.3 mm; see Fig. 5B). The distance to the cerebellar motor representations for all assessed coordinates is presented in Supplementary Figure 1. Notably, the number of subjects where the occipital cortex was closer than the cerebellum was greatest for positions at the level of the inion, and reduced as the displacement from the inion increased in both the lateral and inferior directions (see Fig. 5C). With respect to the two often-used cerebellar stimulation sites, there was a relatively high probability of the shortest distance to cerebellar tissue passing through the occipital cortex at 3L (31/100 subjects), while this probability was considerably reduced at 3L1I (7/100 subjects).

## Discussion

Cerebellar function is increasingly investigated with neurostimulation techniques such as TMS [1]. However, a wide range of different practices exists, with different laboratories using different coil geometries and stimulation protocols [6], [7], [8], [9], [10], [11], [12], [13], [14], [15], [16], [17], [18], [19], [20]. While modeling evidence indicates that coil geometry and stimulation intensity have a considerable impact on the efficacy of TMS stimulation, very little research has addressed the relative merits of different coil geometries in vivo [10]. Here, we are the first to compare the relative efficacy of the figure-of-eight coil, the batwing coil, and the double-cone coil in vivo.

Experiment 1 demonstrated differences in the input–output properties of the three coils when used to stimulate the motor hotspot in M1. Specifically, the batwing and double-cone coils required lower intensities of input stimulus to achieve RMT. Furthermore, increasing stimulus intensity by the same magnitude had greater effects for the batwing and double-cone coils than for the figure-of-eight coil. Note, however, if intensities are considered relative to corresponding coil-specific motor threshold, no significant differences between the slopes for these three coils remain [31]. Our results indicate that coil choice has considerable impact on the relation between the input intensity and the strength of stimulation, but they support the common practice of stimulating other cortical targets at a intensity defined relative to motor threshold [21], [32]. While such a discrepancy has previously been proposed between the batwing and figure-of-eight coils [4], this is the first study to directly compare input–output characteristics in the same subjects.

Experiment 2 systematically investigated the ability of these three TMS coils to elicit CBI, a proxy for cerebellar stimulation. Coil design has a significant effect on the ability to successfully stimulate the cerebellum. The figure-of-eight coil was not able to elicit reliable CBI, even at high values of MSO. The batwing coil elicited CBI only at relatively high values of MSO (75 and 80%), while the double-cone coil elicited CBI at all intensities examined. These results are consistent with Werhahn et al.'s [10] reports that CBI can be reliably elicited with a double-cone, but not with a figure-of-eight coil. Stimulation with the double-cone and batwing coils was deemed more uncomfortable than with the figure-of-eight coil. However, only the double-cone coil was capable of producing reliable CBI at the lower stimulus intensities. Essentially, only the batwing and double-cone coils (both designed to target deep structures), proved reliable in eliciting CBI. Notably, stimulus conditions that induce CBI (Fig. 2) are separated from those that do not by a boundary at a moderate discomfort level of 3.5/7 (dashed line, Fig. 4).

Experiment 3 compared the distance between the scalp and gray matter at the primary motor cortex hand representation and several cerebellar coordinates. Results showed that cerebellar tissue is about one and a half times as far from the scalp than M1 tissue. The distance to the cerebellar hand representations in Lobules V and Lobule VIII (the presumed targets for sensorimotor cerebellar stimulation) are even further removed (30–35 mm). Notably, previous studies indicate that TMS pulses must stimulate beyond the target surface in order to evoke responses [33], [34]. Our estimates can therefore be considered to be conservative, as we calculated the minimum distances from the scalp to the pial surface of the target tissue. Interestingly, our analyses indicated that two scalp coordinates frequently used to stimulate the lateral cerebellum (3L, 3 cm lateral to the inion, and 3L1I, 3 cm lateral and 1 cm inferior of the inion) are amongst the closest distances to cerebellar gray matter. However, scalp coordinates at the same latitude as the inion (e.g. 3L) had a closer distance to occipital tissue than to cerebellar gray matter in a considerable proportion of subjects. Studies that could be confounded by stimulation of the visual cortex should consider this when determining a cerebellar stimulation site.

As the figure-of-eight coil was not able to elicit reliable CBI at any of the intensities used here, it is perhaps surprising that this coil has frequently been used to deliver cerebellar stimulation to the motor system [15], [17]. It is notable that the intensity needed to elicit CBI (and, presumably, to reach cerebellar motor representations) is likely greater than that needed to stimulate more superficial, posterior cerebellar targets. Moreover, as in neocortical tissue, rTMS effects may be present at intensities that are sub-threshold for single pulse stimulation ([35], [36]; though note both papers indicate subthreshold rTMS is less effective than suprathreshold rTMS). Nevertheless, given the distance between the scalp and cerebellar motor representations, it should be considered that the effects reported in previous papers may have been due to the stimulation of more posterior regions of the cerebellum. Future investigations should be careful to account for the depth of cerebellar tissue, for example, by using a depth-adjusted motor threshold [2], [3], [4], [14], [15]. An important factor here is the targeted region of the cerebellum, which could be reasonably superficial in studies of timing [18], working memory [37] and language [12], but relatively deep for studies of motor control.

## Conclusions

This study is the first to compare the in-vivo effects of different coils on cerebellar TMS. Collectively, the results favor the double-cone coil for cerebellar stimulation. Of the three coils compared, the double-cone coil was shown to have the most efficient input–output profile over M1. Using CBI, we were able to determine that only the double-cone coil could effectively stimulate the cerebellum at relatively low stimulus intensities, and was therefore able to produce reliable effects while minimizing discomfort. Tissue depth analyzes indicate that the effectiveness of the double cone coil could in part be explained by the deep position of the cerebellum. The double-cone coil is specifically designed to achieve greater depth of stimulation, making it an appropriate tool to target the deep lying cerebellum and its motor representations. It should be remembered that this increased depth of stimulation is likely to come at the expense of focality [5]. However, the results of the present study demonstrate that the double-cone coil provides a pragmatic solution for effective cerebellar stimulation. These results highlight important considerations for the interpretation of the current literature and the design of future cerebellar TMS experiments.

## References

[1] Grimaldi G., Argyropoulos G.P., Boehringer A. (2014). Non-invasive cerebellar stimulation – a consensus paper. Cerebellum.

[2] Stokes M.G., Chambers C.D., Gould I.C. (2005). Simple metric for scaling motor threshold based on scalp-cortex distance: application to studies using transcranial magnetic stimulation. J Neurophysiol.

[3] Stokes M.G., Chambers C.D., Gould I.C. (2007). Distance-adjusted motor threshold for transcranial magnetic stimulation. Clin Neurophysiol.

[4] Cai W., George J.S., Chambers C.D. (2012). Stimulating deep cortical structures with the batwing coil: how to determine the intensity for transcranial magnetic stimulation using coil-cortex distance. J Neurosci Methods.

[5] Deng Z.D., Lisanby S.H., Peterchev A.V. (2013). Electric field depth-focality tradeoff in transcranial magnetic stimulation: simulation comparison of 50 coil designs. Brain Stimul.

[6] Galea J.M., Jayaram G., Ajagbe L., Celnik P. (2009). Modulation of cerebellar excitability by polarity-specific noninvasive direct current stimulation. J Neurosci.

[7] Ugawa Y., Uesaka Y., Terao Y., Hanajima R., Kanazawa I. (1995). Magnetic stimulation over the cerebellum in humans. Ann Neurol.

[8] Pinto A.D., Chen R. (2001). Suppression of the motor cortex by magnetic stimulation of the cerebellum. Exp Brain Res.

[9] Daskalakis Z.J., Paradiso G.O., Christensen B.K. (2004). Exploring the connectivity between the cerebellum and motor cortex in humans. J Physiol.

[10] Werhahn K.J., Taylor J., Ridding M., Meyer B.U., Rothwell J.C. (1996). Effect of transcranial magnetic stimulation over the cerebellum on the excitability of human motor cortex. Electroencephalogr Clin Neurophysiol.

[11] Fisher K.M., Lai H.M., Baker M.R., Baker S.N. (2009). Corticospinal activation confounds cerebellar effects of posterior fossa stimuli. Clin Neurophysiol.

[12] Lesage E., Morgan B.E., Olson A.C., Meyer A.S., Miall R.C. (2012). Cerebellar rTMS disrupts predictive language processing. Curr Biol.

[13] Miall R.C., King D. (2008). State estimation in the cerebellum. Cerebellum.

[14] Panouillères M., Neggers S.F., Guttelin T.P. (2012). Transcranial magnetic stimulation and motor plasticity in human lateral cerebellum: dual effect on saccadic adaptation. Hum Brain Mapp.

[15] Popa T., Russo M., Meunier S. (2010). Long-lasting inhibition of cerebellar output. Brain Stimul.

[16] Demirtas-Tatlidede A., Freitas C., Pascual-Leone A., Schmahmann J.D. (2011). Modulatory effects of theta burst stimulation on cerebellar nonsomatic functions. Cerebellum.

[17] Koch G., Mori F., Marconi B. (2008). Changes in intracortical circuits of the human motor cortex following theta burst stimulation of the lateral cerebellum. Clin Neurophysiol.

[18] Théoret H., Haque J., Pascual-Leone A. (2001). Increased variability of paced finger tapping accuracy following repetitive magnetic stimulation of the cerebellum in humans. Neurosci Lett.

[19] Grube M., Lee K.H., Griffiths T.D., Barker A.T., Woodruff P.W. (2010). Transcranial magnetic theta-burst stimulation of the human cerebellum distinguishes absolute, duration-based from relative, beat-based perception of subsecond time intervals. Front Psychol.

[20] Miall R.C., Christensen L.O.D., Cain O., Stanley J. (2007). Disruption of state estimation in the human lateral cerebellum. PLoS Biol.

[21] Hallett M. (2007). Transcranial magnetic stimulation: a primer. Neuron.

[22] Guzmán-López J., Costa J., Selvi A. (2012). The effects of transcranial magnetic stimulation on vibratory-induced presynaptic inhibition of the soleus H reflex. Exp Brain Res.

[23] Biswal B.B., Mennes M., Zuo X.N. (2010). Toward discovery science of human brain function. Proc Natl Acad Sci U S A.

[24] Kleim J.A., Kleim E.D., Cramer S.C. (2007). Systematic assessment of training-induced changes in corticospinal output to hand using frameless stereotaxic transcranial magnetic stimulation. Nat Protoc.

[25] Rosenkranz K., Williamon A., Rothwell J.C. (2007). Motorcortical excitability and synaptic plasticity is enhanced in professional musicians. J Neurosci.

[26] Zhang X., de Beukelaar T.T., Possel J. (2011). Movement observation improves early consolidation of motor memory. J Neurosci.

[27] Hardwick R.M., McAllister C.J., Holmes P.S., Edwards M.G. (2012). Transcranial magnetic stimulation reveals modulation of corticospinal excitability when observing actions with the intention to imitate. Eur J Neurosci.

[28] Diedrichsen J. (2006). A spatially unbiased atlas template of the human cerebellum. Neuroimage.

[29] Diedrichsen J., Balsters J.H., Flavell J., Cussans E., Ramnani N. (2009). A probabilistic MR atlas of the human cerebellum. Neuroimage.

[30] Buckner R.L., Krienen F.M., Castellanos A., Diaz J.C., Yeo B.T.T. (2011). The organization of the human cerebellum estimated by intrinsic functional connectivity. J Neurophysiol.

[31] Peterchev A.V., Goetz S.M., Westin G.G., Luber B., Lisanby S.H. (2013). Pulse width dependence of motor threshold and input-output curve characterized with controllable pulse parameter transcranial magnetic stimulation. Clin Neurophysiol.

[32] Loporto M., McAllister C., Williams J., Hardwick R., Holmes P. (2011). Investigating central mechanisms underlying the effects of action observation and imagery through transcranial magnetic stimulation. J Mot Behav.

[33] Epstein C.M., Schwartzberg D.G., Davey K.R., Sudderth D.B. (1990). Localizing the site of magnetic brain stimulation in humans. Neurology.

[34] Marg E., Rudiak D. (1994). Phosphenes induced by magnetic stimulation over the occipital brain: description and probable site of stimulation. Optom Vis Sci.

[35] Fitzgerald P.B., Brown T.L., Daskalakis Z.J., Chen R., Kulkarni J. (2002). Intensity-dependent effects of 1 Hz rTMS on human corticospinal excitability. Clin Neurophysiol.

[36] Lang N., Harms J., Weyh T. (2006). Stimulus intensity and coil characteristics influence the efficacy of rTMS to suppress cortical excitability. Clin Neurophysiol.

[37] Desmond J.E., Chen S.H.A., Shieh P.B. (2005). Cerebellar transcranial magnetic stimulation impairs verbal working memory. Ann Neurol.

