# Supplementary material for: Cerebellar Transcranial Magnetic Stimulation: The Role of Coil Geometry and Tissue Depth
Source: Brain Stimul. 2014 Sep;7(5):643–9. doi: 10.1016/j.brs.2014.04.009 (PMC4180011; doi:10.1016/j.brs.2014.04.009)
Supplement: Supplementary Material [file mmc1.doc]

**Supplementary Materials**

**Supplementary Methods**

***Experiment 1***

*Electromyography*

Electromyograms were recorded from the first dorsal interosseus (FDI) muscle of the dominant hand using a bipolar electrode configuration. A common reference electrode was positioned over the ulnar styloid process at the wrist. The signal was bandpass filtered from 20 to 1000Hz using a 1902 amplifier (Cambridge Electronic Design, Cambridge, UK). The resulting electromyogram was digitized with a sampling rate of 2kHz using a micro CED 1401 A-D convertor with Signal (version 4) software (both by CED). MEPs were recorded from the dominant hand and elicited via stimulation of the contralateral M1.

*Motor Hotspot & Resting Motor Threshold*

The motor hotspot was defined as the position over the primary motor cortex (M1) from which the largest motor evoked potentials (MEPs) from the FDI muscle were observed. The hotspot was assessed separately for each coil, but was found to correspond to the same position regardless of coil design. The motor hotspot position was then recorded using a frameless stereotaxic system (Brainsight 2, Rogue Research, Montreal, Canada). Resting motor threshold (RMT) was defined as the lowest stimulation intensity to elicit MEPs with peak-to-peak amplitudes greater than 50µV in 50% (5/10) of trials. EMG activity 100ms before the stimulus was monitored in order to determine that the hand was at rest. In experiment 1, RMT was measured using the figure-of-eight, batwing and double-cone coils separately, and recorded as a percentage of maximal stimulator output (MSO).

***Experiment 2***

*Control for corticospinal tract stimulation*

Stimulation of the cerebellum can be confounded by ‘direct’ corticospinal tract (CST) stimulation (e.g. stimulation of the CST at the level of the brainstem; Ugawa et al., 1995). The presence of direct CST activation can be assessed by monitoring the latency of the first EMG response above baseline (Fisher et al, 2009), a method that has been shown to be more sensitive than intensity calibration based on measures of direct motor threshold used in previous studies (Ugawa et al, 1995; Matsunaga et al, 2001). Short latency EMG responses for conditioned pulses (i.e. cerebellar and M1 stimulation) in comparison to the control pulses (i.e. M1 stimulation alone) indicate the presence of direct CST activity. We therefore conducted a 3x4x2 repeated measures ANOVA on first EMG response latency to TMS. EMG response latency was assessed by scanning EMG traces for the first deflection above a 95% confidence interval for the median baseline EMG.

***Experiment 3***

*Cerebellar depth*

A sample of 100 T1 structural scans (Buckner et al., 2010) were used to assess the average distance from the scalp to targeted neuronal tissue in the cerebellum and the hand representation in primary motor cortex. Each structural scan was used to create a volume containing voxels that identified tissue as either the head, grey matter, cerebellar grey matter, cerebellar hand representations, or the motor cortex hand representation. The volume for each participant was generated in a five step process. First, the structural scan was binarized to leave only an outline of the head. Second, a grey matter mask of the structural scan was created using the SPM5 segmentation algorithm. Third, cerebellar tissue was isolated using the SUIT toolbox (Diedrichsen, 2006). Fourth, clusters identified at the group in fMRI that correspond to the cerebellar and motor cortex hand representations (Buckner et al., 2011) were warped into the individual subjects’ native space. To achieve this, normalization parameters from the native space to the MNI template brain were calculated with SPM5 normalization (Ashburner & Friston, 2005). The inverse normalization parameters were then applied to the hand representation clusters in M1 and the cerebellum. In the fifth and final step, the volumes created in the previous steps were binarized, given numeric codes, and combined into the final volume. Thus, the resulting final volumes were spatially coded in native space, with voxels that had different intensity values for tissue corresponding to the head, grey matter, the cerebellum, the superior hand representation in the cerebellum, the inferior hand representation in the cerebellum, and the hand representation in the motor cortex. All binarizations were performed with the fslmaths algorithms in the FMRIB software library (<http://fsl.fmrib.ox.ac.uk/fsl/fslwiki/>).

Previous TMS studies have stimulated the cerebellum based on positioning the coil over scalp coordinates spaced relative to the inion (c.f. Ugawa et al., 1994). Here we determined the position of the inion for each subject, and used a custom matlab script (the Mathworks company) to generate a grid of 25 scalp positions relative to the inion, moving 3cm inferior and 4cm lateral (right) in 1cm steps (see Figure S1). For each grid position we calculated the shortest Cartesian distance to cerebellar gray matter, and to clusters corresponding to the inferior and superior cerebellar hand representations (Buckner et al., 2011). For comparison, we also computed the shortest distance between the scalp and to a cluster corresponding to the hand representation in the primary motor cortex.

Because of the proximity of superior portions of the cerebellum to the occipital lobe, we also calculated the likelihood of stimulating non-cerebellar (occipital) tissue. This was defined as the probability that non-cerebellar grey matter was more than 2 mm closer to the scalp location than cerebellar grey matter.

**Supplementary Results**

***Experiment 2***

*Control for brainstem stimulation effects*

Direct CST triggered MEPs occur at shorter latencies than responses elicited from M1 (Fisher et al., 2009). We therefore tested for direct CST activation by comparing EMG activity onset latencies for all CBI conditions against their corresponding control condition. Response latencies were submitted to a 3x4x2 ANOVA. This analysis revealed no significant main effect or interaction effects of cerebellar conditioning pulses on response latencies (all F<1.5, p>0.25). Thus, there is no evidence for the shorter MEP latencies that would indicate brainstem stimulation.

***Experiment 3***

*Cerebellar depth*

*
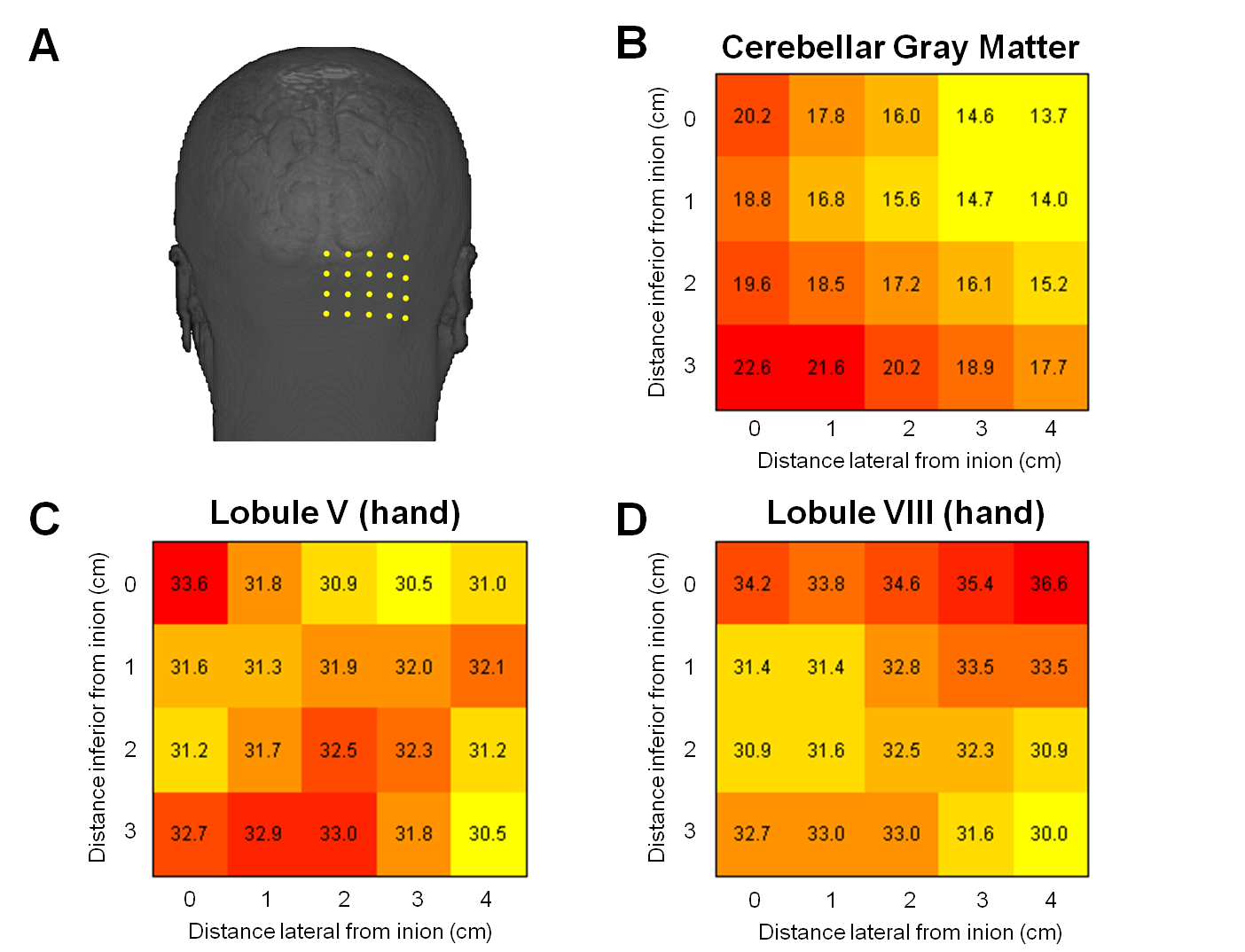
*

*Supplementary Figure 1: Average distances from scalp coordinates on the 5x4 grid to cerebellar tissue. Distances are reported in mm.*

As show in supplementary figure 1, the absolute distance to cerebellar grey matter tissue was shortest for 3 (3L;14.6 +/- 2.6 mm) and 4 cm lateral to the inion (4L; 13.7 +/- 2.8 mm), and for the same locations 1 cm inferior to the inion (3L1I: 14.7 +/- 3.6 mm;4L1I: 14.0 +/- 3.3mm). Distances were notably longer for the two cerebellar motor representations. The lobule V representation was closest to locations 3L (30.5 +/- 4.0mm) and 4L3I (30.5 +/- 5.0mm), and distances from these coordinates did not differ significantly from one another (difference = 0.04mm; t_99_ = 0.09, p = 0.924). To the Lobule VIII motor representation, locations more inferior from the inion were closer than the often-used coordinates 3L and 3L1I, with the closest locations those directly below to the inion, and on the lateral and inferior portions of the grid. Note that on average, the Lobule VIII hand representation was even deeper than the Lobule V hand representation, such that the closest position to the inferior hand representation (4L3I) was not much closer to the Lobule VIII representation (30.0 +/-4.8mm) than to the Lobule V representation (30.5 +/- 5.0mm), though the difference was significant (difference = 0.43mm; t_99_ = 2.333, p<0.022). That is, a TMS pulse that reaches the VIII representation might also reach the Lobule V representation.
